# Exploring the shared molecular mechanisms of primary hypertension and IgA vasculitis through a case report and combining bioinformatics analysis

**DOI:** 10.3389/fimmu.2025.1596174

**Published:** 2025-06-06

**Authors:** Qijiao Wei, Jiayan Feng, Yifei Mu, Qian Shen, Hong Xu, Li Sun, Haimei Liu

**Affiliations:** ^1^ Department of Rheumatology, Children’s Hospital of Fudan University, National Pediatric Medical Center of China, Shanghai, China; ^2^ Department of Pathology, Children’s Hospital of Fudan University, National Pediatric Medical Center of China, Shanghai, China; ^3^ Department of Pediatric, The First People’s Hospital of Zunyi, The Third Affiliated Hospital of Zunyi Medical University, Zunyi, China; ^4^ Department of Nephrology, Children’s Hospital of Fudan University, National Pediatric Medical Center of China, Shanghai, China

**Keywords:** primary hypertension, IgA vasculitis, blood pressure-lowering agents, bioinformatics analysis, differentially expressed genes

## Abstract

**Background:**

Primary hypertension (PHTN) and IgA vasculitis (IgAV) are two prevalent medical conditions that affect the kidneys. Despite their distinct pathophysiological mechanisms, clinical manifestations, and treatment approaches, both conditions significantly impact patients’ quality of life and overall health. Unfortunately, a boy developed hypertensive nephritis (HN) at the age of 4, followed by IgAV nephritis at the age of 10. The exact pathophysiological mechanisms underlying these conditions remain elusive. This study aims to report this unusual case and investigate the biological mechanisms associated with the differentially expressed genes (DEGs) related to PHTN and IgAV through the application of bioinformatics tools.

**Methods:**

We present the case of a boy diagnosed with PHTN and IgAV. Additionally, we explore the molecular mechanisms underlying both conditions. DEGs were analyzed, and gene functional enrichment was performed using the DAVID database. A protein-protein interaction (PPI) network was constructed using the STRING database and visualized with Cytoscape software. The hub genes were identified using the MCODE plugin. CIBERSORT was used to assess the expression changes in immune cells and obtain the proportion of various types of immune cells. Furthermore, the Connectivity Map L1000 platform was utilized to identify potential therapeutic agents.

**Results:**

The boy initially presented with malignant hypertension (MHT), and renal biopsy pathology indicated HN. Following regular use of antihypertensive medications, there was a significant improvement in blood pressure (BP), renal function, and the left ventricular hypertrophy index. However, he developed IgAV six years after the diagnosis of PHTN. A subsequent renal biopsy revealed IgAV nephritis, and the pathology associated with HN showed marked improvement. A total of 7,027 DEGs associated with PHTN and 90 DEGs linked to IgAV were identified, with 25 genes overlapping between the two sets. KEGG pathway analysis revealed that the DEGs were primarily associated with extracellular matrix (ECM) receptor interaction. Among EMC key components, fibronectin expression was markedly elevated in hypertensive nephritis and IgA nephropathy. Gene Ontology (GO) biological process analysis indicated that the 25 overlapping DEGs were significantly related to processes such as proteolysis, amyloid fibril formation, cAMP-mediated signaling, synaptic vesicle endocytosis, and receptor internalization. The significantly enriched terms related to changes in the cellular component of DEGs included platelet alpha granule membrane, nucleoplasm, and endocytic vesicle membrane. Changes in molecular function were primarily associated with protein binding. Furthermore, six hub genes implicated in both diseases were linked to cell adhesion molecules. We also found that neutrophils accounted for the majority of all infiltrating cells. And B cell naïve were downregulated in both diseases.Using the Connectivity Map (CMap) database, the top 10 potential therapeutic agents were identified.

**Conclusion:**

We found that aggressive BP-lowering agents were necessary for managing PHTN. This study also reveals the common pathogenesis underlying both PHTN and IgAV. Moving forward, these shared hub genes could serve as novel targets for more in-depth mechanistic investigations and the development of new therapeutic interventions for individuals affected by PHTN and IgAV.

## Highlights

- Aggressive blood pressure-lowering agents are necessary for managing PHTN.
- The mechanisms underlying the DEGs associated with PHTN and IgAV were explored for the first time.
- Extracellular matrix (ECM) receptor interaction participated in the pathogenesis of PHTN and IgAV.
- Fibronectin expression was markedly elevated in hypertensive nephritis and IgA nephropathy.
- We identified several compounds as potential treatment options for PHTN and IgAV.

## 1 Introduction

Primary hypertension (PHTN) and IgA vasculitis (IgAV) are two prevalent medical conditions that affect the kidneys. PHTN is a chronic condition characterized by consistently elevated blood pressure without an identifiable secondary cause (1). Hypertensive nephropathy (HN) refers to kidney damage resulting from prolonged high blood pressure (BP) (2). Genetic predisposition and environmental factors play significant roles in the development of PHTN (3). Several physiological mechanisms contribute to the onset of PHTN, including renal mechanisms, sympathetic nervous system activation, and endothelial dysfunction (4). HN develops as a consequence of sustained high blood pressure, leading to structural and functional changes in the kidneys. The cumulative effect of these changes results in decreased glomerular filtration rate (GFR), proteinuria, and ultimately chronic kidney disease (CKD) (5). In contrast, IgA vasculitis (IgAV), also known as Henoch-Schönlein purpura (HSP), is a small-vessel vasculitis characterized by a tetrad of symptoms: palpable purpura, often on the buttocks and legs, arthralgia or arthritis, abdominal pain, and renal involvement (6). IgAV is thought to be related to an abnormal immune response (7), with key features including IgA deposition and vascular inflammation (8).

Despite their divergent pathophysiological origins, we encountered a rare case of concurrent PHTN and IgAV in a pediatric patient. This clinical overlap raises intriguing questions about potential shared molecular mechanisms. Understanding these common pathways and mechanisms is crucial for advancing treatment strategies. This study aims to present this remarkable case and delve into the biological mechanisms that govern the differentially expressed genes (DEGs) associated with PHTN and IgAV. By employing advanced bioinformatics tools, we seek to uncover the intricate relationships and underlying pathways that contribute to these conditions.

## 2 Methods

### 2.1 Case report

This study was approved by the Institutional Review Board of Children’s Hospital of Fudan University (No. 2021-169), and informed consent was obtained from the participant. We collected demographic, clinical, laboratory characteristics, and follow-up information for the patients included in this study. We report the clinical manifestations, diagnostic processes, and prognosis of the patient.

### 2.2 Microarray data

NCBI-GEO (http://www.ncbi.nlm.nih.gov/geo) serves as a centralized repository for high-throughput functional genomics data, including microarray and RNA sequencing datasets (9). We conducted a search using the keywords “primary hypertension” OR “essential hypertension,” which yielded 51 results in the GEO Database. After filtering for Homo sapiens, 27 results remained. We carefully examined these 27 entries. In this study, we aim to explore the differentially expressed genes (DEGs) between patients with PHTN and normal controls, ultimately identifying only GSE24752 for further analysis (10). Additionally, we searched using the keywords “IgA vasculitis,” which returned 14 results in the GEO Database. Our goal is to investigate the DEGs between IgAV patients and normal controls, leading us to focus on GSE102114 (11).

### 2.3 Identification of DEGs

The GEO2R online tool was utilized for the identification of DEGs (9). A log fold change (FC) greater than 1 and a P-value less than 0.05 were considered statistically significant. The raw data were analyzed using Venn software to identify common DEGs. DEGs with a log FC less than 0 were classified as downregulated, while those with a log FC greater than 0 were classified as upregulated.

### 2.4 Functional enrichment and PPI analysis

The Database for Annotation, Visualization, and Integrated Discovery (DAVID; http://david.ncifcrf.gov) is an online biological information resource (12). The Kyoto Encyclopedia of Genes and Genomes (KEGG) serves as a database for understanding high-level functions and biological systems derived from large-scale molecular datasets generated by high-throughput experimental technologies (13). Gene Ontology (GO) is a key bioinformatics tool used for annotating genes and analyzing their biological processes (14). In this study, DAVID 6.8 Bioinformatics Resources was employed for pathway annotations, with statistical significance set at P < 0.05. A protein-protein interaction (PPI) network was constructed using the Search Tool for the Retrieval of Interacting Genes (STRING) (version 10.0) available at https://string-db.org/, with interactions exhibiting a combined score greater than 0.4 considered statistically significant. The PPI network was visualized using Cytoscape (version 3.7.2) (https://cytoscape.org/).

### 2.5 Profiling infiltrating immune cells with CIBERSORT

To systematically evaluate compositional alterations in immune cell populations and quantify subtype-specific lymphocyte proportions, we employed the web-based CIBERSORT analytical tool (https://cibersort.stanford.edu/). This machine learning approach, based on deconvolution principles, enables precise characterization of immune cell fractions through linear support vector regression analysis of gene expression data. Comparative analyses were conducted using a standardized leukocyte gene signature matrix (LM22) that distinguishes 22 functionally distinct immune cell subtypes. The analytical workflow incorporated 1000 permutations with p-value thresholding (p<0.05) to ensure statistically robust cell fraction estimations.

### 2.6 CMap analysis

The Connectivity Map (CMap) was utilized to identify potential agents for PHTN and IgAV. The 25 DEGs were queried using the Connectivity Map online tool (L1000 platform; https://clue.io/l1000-query) (15). Upon submitting a list of 20 upregulated genes, connectivity scores ranging from -1 to 1 were generated to reflect the similarity between expression profiles. A positive score indicates a promoted effect, while a negative score signifies an inhibited effect.

### 2.7 Pathological findings of renal biopsies

The renal biopsy specimens were embedded in OCT compound (Sakura, Hayward, CA, USA) and paraffin wax, respectively, to prepare frozen sections and paraffin sections. Routine pathological staining methods, including hematoxylin-eosin (HE) staining and immunofluorescence staining, were performed on the sections. Furthermore, Masson’s trichrome staining was conducted on the renal tissue sections, and immunofluorescence staining for Fibronectin was employed to observe its expression pattern in renal tissues.

## 3 Result

### 3.1 Case presentation

In April 2012, a 4-year-old boy presented to a local hospital with recurrent dizziness for 3 weeks. Initial evaluation revealed severe hypertension. BP was 210/150 mmHg. After treatment with sodium nitroprusside, nifedipine, phentolamine, and benazepril, his BP was maintained at 130-150/72-90 mmHg. However, subsequent nonadherence to the prescribed regimen led to uncontrolled hypertension, prompting referral to the Children’s Hospital of Fudan University on October 13, 2012, for specialized evaluation and management.

Physical examination demonstrated marked hypertension (BP: 140/100 mmHg (>99th percentile for age/height: 118/77 mmHg). Anthropometric measurements revealed normal growth parameters: height 107 cm (50th percentile), weight 16.6 kg (25th-50th percentile), and BMI was 14.4 kg/m². Neurological assessment showed an alert, interactive child with no signs of encephalopathy (e.g., seizures, altered consciousness) or volume overload (e.g., edema, oliguria). Nutritional status and developmental milestones were age-appropriate, with preserved appetite. No significant pre-existing medical conditions were documented. However, familial medical history remained unavailable due to the patient’s adoptive status.

Laboratory investigations ruled out secondary hypertension etiologies: autoantibodies including ANA, ANCA, anti-dsDNA returned negative, and endocrine profiles including thyroid function, cortisol, renin-angiotensin-aldosterone system were within normal ranges. Renal involvement was evidenced by proteinuria (++), microscopic hematuria (3–4 RBCs/high-power field), and elevated urinary protein excretion (urine protein/creatinine ratio: 0.97–1.13; 24-hour urine protein: 0.32 g). Blood biochemistry demonstrated preserved renal function (serum creatinine: 46.0 µmol/L) and normoalbuminemia (43.4 g/L). Histopathological analysis of renal biopsy specimens confirmed malignant nephrosclerosis, characterized by arteriolar fibrinoid necrosis and ischemic glomerular collapse. Advanced imaging studies excluded structural anomalies: Normal brain and pituitary gland on MRI (plain + contrast-enhanced sequences). Vascular imaging: No renal artery stenosis (CTA), urinary tract malformations (MCU), or vascular lesions (DSA). Cardiac evaluation revealed left ventricular hypertrophy with preserved systolic function (left ventricular ejection fraction: 77%; cardiac output: 3.56 L/min). Electrocardiographic findings included left axis deviation and left ventricular high voltage, consistent with pressure overload. Ophthalmologic assessment ruled out hypertensive retinopathy, with fundoscopic examination showing no vascular abnormalities.

Secondary hypertension etiologies—including connective tissue disorders, endocrine pathologies (hyperthyroidism, Cushing’s syndrome, pheochromocytoma), Takayasu arteritis, and CNS tumors—were systematically excluded based on comprehensive laboratory and imaging findings. The patient was thus diagnosed with PHTN complicated by HN. Antihypertensive therapy with felodipine, benazepril, and metoprolol was initiated, achieving blood pressure stabilization (100–110/60–70 mmHg) by discharge on November 6, 2012. From 2013 to 2017, strict adherence to the regimen maintained normotension. Serial monitoring demonstrated preserved renal function (normal urinalysis, serum creatinine) and resolved cardiac remodeling (normalized ECG and echocardiographic parameters).

In October 2017, when the boy was 9 years old, he developed purpura. prompting a diagnosis of IgA vasculitis (IgAV) at a local hospital, with symptom resolution following undocumented treatment. Post-discharge renal monitoring (urinalysis, creatinine) was neglected. By August 2018, at the age of 10, he re-presented with systemic symptoms (vomiting, diarrhea, headache, dizziness) and nephritic-nephrotic syndrome features: gross hematuria, foamy urine, nephrotic-range proteinuria (urine protein+++, UPCR 8.73, 24-hour protein 5.38–6.04 g), hypoalbuminemia (27.3 g/L), and elevated creatinine (115 µmol/L). Renal biopsy confirmed IgAV nephritis (ISKDC grade IVb), prompting pulse methylprednisolone (1 g/day ×3) and cyclophosphamide induction, followed by oral prednisone, alongside antihypertensive optimization (felodipine/metoprolol continued; benazepril halted due to renal impairment). Post-therapy urinalysis showed persistent proteinuria (+++) and microscopic hematuria (36 RBCs/hpf), with stabilized BP (100–110/60–70 mmHg) prior to discharge on September 20, 2018.

According to our hospital’s post-treatment evaluation records, only four weeks since the initial treatment. And the patient exhibited no clinically significant improvement (**Table 1**). Consequently, the child was transferred to a local medical institution for continued care management. Unfortunately, the patient was lost to follow-up after discharge. Due to the sensitive nature of the patient’s adoptive status, the guardians declined to provide additional demographic or clinical details beyond mandatory reporting requirements. And his current condition remains unknown.

**Table 1:** Clinical and laboratory information of the boy at different stages of the disease.

Term	PHTN		IgAV	
	At onset 2012.05	After treatment 2016.07	At onset 2018.08	After treatment 2018.09
Age (years)	4	8	10	10
White blood cells (WBC, ×10^9^/L)	8.5	6.6	7.8	25.6
Hemoglobin (HB, g/L)	153	130	140	137
Platelets (PLT, ×10^9^/L)	415	276	473	535
Erythrocyte Sedimentation Rate (ESR, mm/h)	NA	NA	29	NA
Albumin (Alb, g/L)	43.4	NA	27.3	25.8
Creatinine (Cr, μmol/L)	46	45	115	117
Blood Urea Nitrogen (BUN, mmol/L)	6.6	6.6	8.7	7.4
Urine protein	2(+)	(-)	3(+)	3(+)
24-hour urine protein (24h-UP, g/24h)	0.32	NA	5.38	6.04
Urinary protein/creatinine	0.97-1.13	NA	8.73	NA
GFR (Renal dynamic imaging)	35.7ml/min (Left: 15.0ml/min, Right: 20.7ml/min)	49.0ml/min (Left: 21.9ml/min, Right: 27.1ml/min)	31.1ml/min (Left: 15.0ml/min, Right: 16.1ml/min)	NA
Renal pathology (See **Table 2**)	Hypertensive Nephropathy (HN)	NA	HN with IgAV nephritis (IVb)	NA
Treatment	Felodipine, Benazepril, and metoprolol	Felodipine, Benazepril, and metoprolol	Methylprednisolone pulse therapy, Cyclophosphamide pulse therapy and Oral Prednisone for IgAV Felodipine and metoprolol for PHTN	Oral Prednisone Felodipine, metoprolol

### 3.2 Renal pathology at different stage

**Figure 1:**
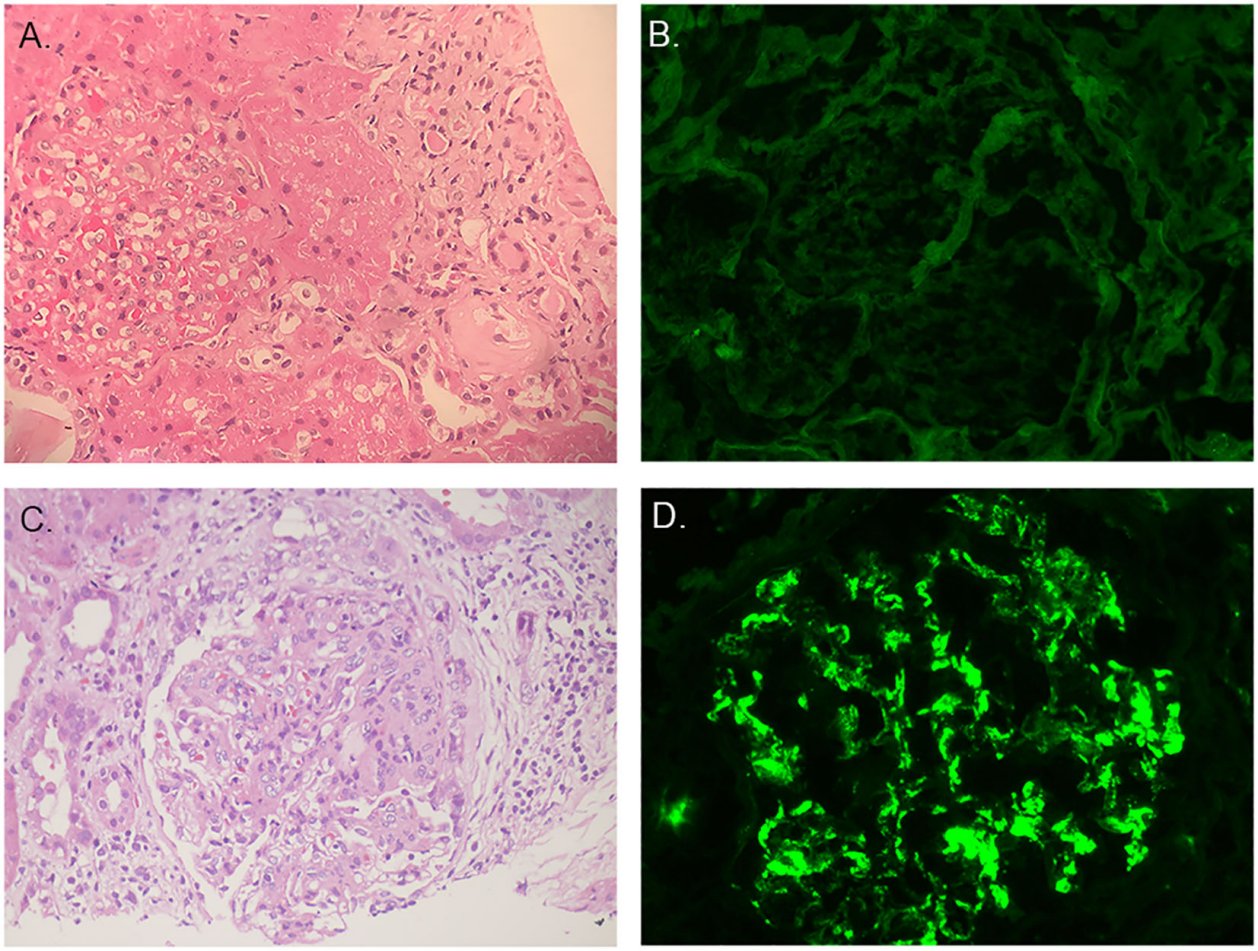
Pathological findings of renal biopsies. **(A)** The first renal biopsy: H&E staining. **(B)** The first renal biopsy: immunofluorescence staining showed IgA was negative. **(C)** The second renal biopsy: H&E staining. **(D)** The second renal biopsy: immunofluorescence staining showed IgA was positive.

In october 2012, the pathologic diagnosis indicated HN. HE staining displayed that seven out of ten glomeruli had glomerulosclerosis, no crescent were seen. Renal tubular had atrophy. Thickening of interstitial vascular wall and interstitial fibrosis were seen and partial blood vessels had hyalinization (**Figure 1A**). Immunofluorescence displayed that IgG(-), IgA(-) (**Figure 1B**), IgM(±), C3(-), C4(-), C1q(-), PLA2-R1(-), CollagenIVα1(+), CollagenIVα3(+), CollagenIVα5(+) (**Table 2**).

**Table 2:** Renal pathology at different stages of the disease.

Term	PHTN (2012.10)	IgAV (2018.09)
Pathologic Diagnosis	Pathological changes of hypertensive nephropathy	Hypertensive nephropathy with IgAV nephritis (IVb)
Glomerulus	Seven out of ten glomeruli had glomerulosclerosis, no crescent	Out of the 23 glomeruli, 7 exhibited glomerulosclerosis, 1 was nearing sclerosis, and the majority showed moderate to severe mesangial cell proliferation accompanied by increased matrix. Some glomerular capillary loops were adherent to the Bowman’s capsule, and 5 cellular crescents were observed.
Renal tubules	Renal tubular atrophy	The proximal tubular epithelial cells exhibited mild swelling with partial vacuolization. Red blood cell casts, cellular casts, and protein casts were observed within the tubular lumens.
Renal interstitium	Thickening of interstitial vascular wall and interstitial fibrosis	Significant inflammatory cell infiltration in the renal interstitium accompanied by interstitial fibrosis
Renal small blood vessels	Partial blood vessels had hyalinization	Some small arterial walls were thickened with accompanying luminal narrowing
Immunofluorescence	IgG(-), IgA(-), IgM(±), C3(-), C4(-), C1q(-), PLA2-R1(-), CollagenIVα1(+), CollagenIVα3(+), CollagenIVα5(+)	IgG(-), IgA(+++), IgM(-), C3(±), C4(-), C1q(-), PLA2-R1(-),CollagenIVα1(+), CollagenIVα3(+), CollagenIVα5(+)

In August 2018, the boy was diagnosed with IgAV and developed hematuria and proteinuria, so a second renal biopsy was performed. The pathologic diagnosis was HN with IgAV nephritis (IVb).

Out of the 23 glomeruli, 7 exhibited glomerulosclerosis, 1 was nearing sclerosis, and the majority showed moderate to severe mesangial cell proliferation accompanied by increased matrix. Some glomerular capillary loops were adherent to the Bowman’s capsule, and 5 cellular crescents were observed. The proximal tubular epithelial cells exhibited mild swelling with partial vacuolization. Red blood cell casts, cellular casts, and protein casts were observed within the tubular lumens. Significant inflammatory cell infiltration in the renal interstitium accompanied by interstitial fibrosis. Some small arterial walls were thickened with accompanying luminal narrowing (**Figure 1C**). Immunofluorescence displayed that IgG(-), IgA(+++) (**Figure 1**), IgM(-), C3(±), C4(-), C1q(-), PLA2-R1(-),CollagenIVα1(+), CollagenIVα3(+), CollagenIVα5(+) (**Table 2**).

### 3.3 DEG identification

**Figure 2:**
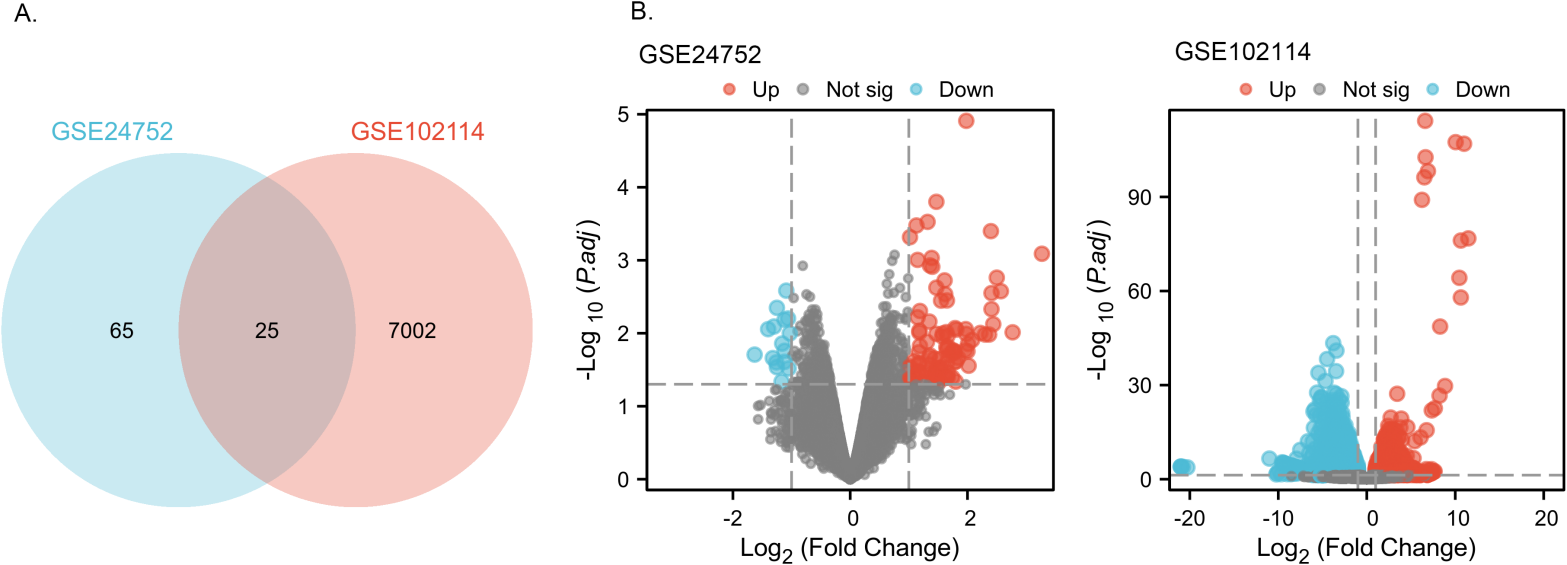
Identification of common DEGs from GSE24752, GSE102114 datasets. **(A)** Venn diagram of DEGs based on the two GEO datasets. **(B)** Volcano plot of the DEGs. Red, upregulation; green, downregulation.

From the GSE24752 dataset, a total of 90 DEGs were successfully identified, comprising 79 upregulated and 11 downregulated genes. In the GSE102114 dataset, we observed 7,027 DEGs, which included 2,600 upregulated and 4,427 downregulated genes. Among all the DEGs identified, 25 were common to both datasets, as illustrated in the Venn diagram (**Figure 2**). These 25 DEGs included 5 downregulated genes and 20 upregulated genes. The DEGs are depicted in **Figure 2**, where red dots represent upregulated genes and green dots represent downregulated genes.

### 3.4 GO annotation and KEGG pathway enrichment analyses

**Figure 3:**
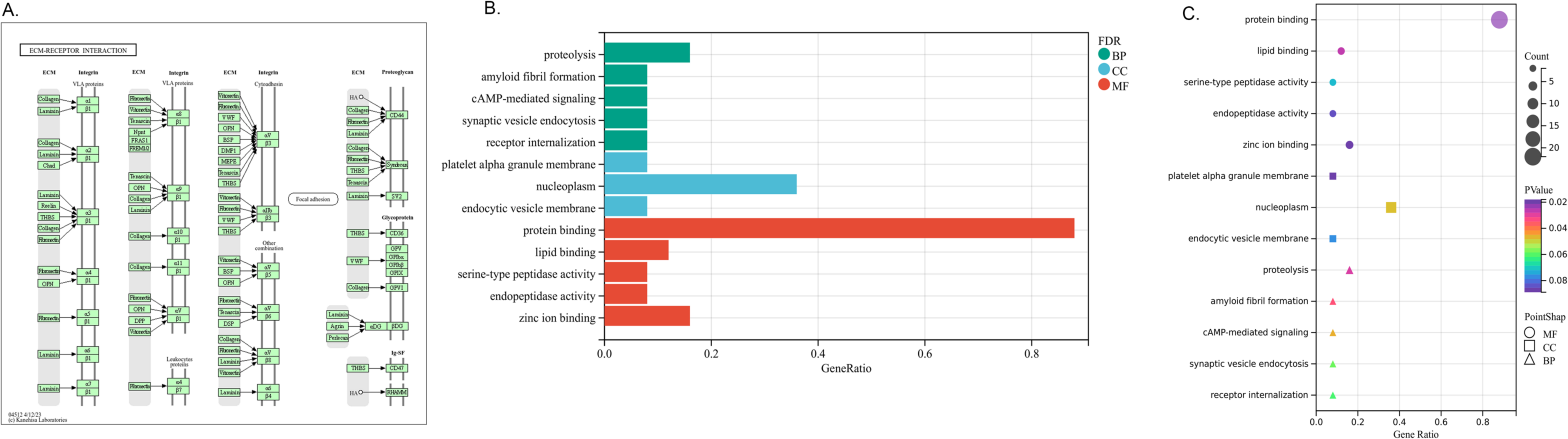
Distribution of integrated DEGs for different enriched functions. **(A)** KEGG pathway- EMC receptor interaction. **(B)** The DEGs enrichment of BP, MF, CC in bar chart. **(C)** The DEGs enrichment of BP, MF, CC in bubble chart.

To gain deeper insight into the biological roles of these 25 DEGs, we conducted functional and pathway enrichment analyses using DAVID. The enriched GO terms and KEGG pathways are presented in **Figure 3**. KEGG pathway analysis revealed that the DEGs were primarily associated with extracellular matrix (ECM) receptor interaction. GO biological process analysis indicated that the 25 DEGs were significantly associated with proteolysis, amyloid fibril formation, cAMP-mediated signaling, synaptic vesicle endocytosis and receptor internalization. The significantly enriched terms regarding changes in cell component of DEGs were platelet alpha granule membrane, nucleoplasm and endocytic vesicle membrane. Changes in molecular function are primarily associated with protein binding.

### 3.5 PPI analysis and infiltrating immune cells

**Figure 4:**
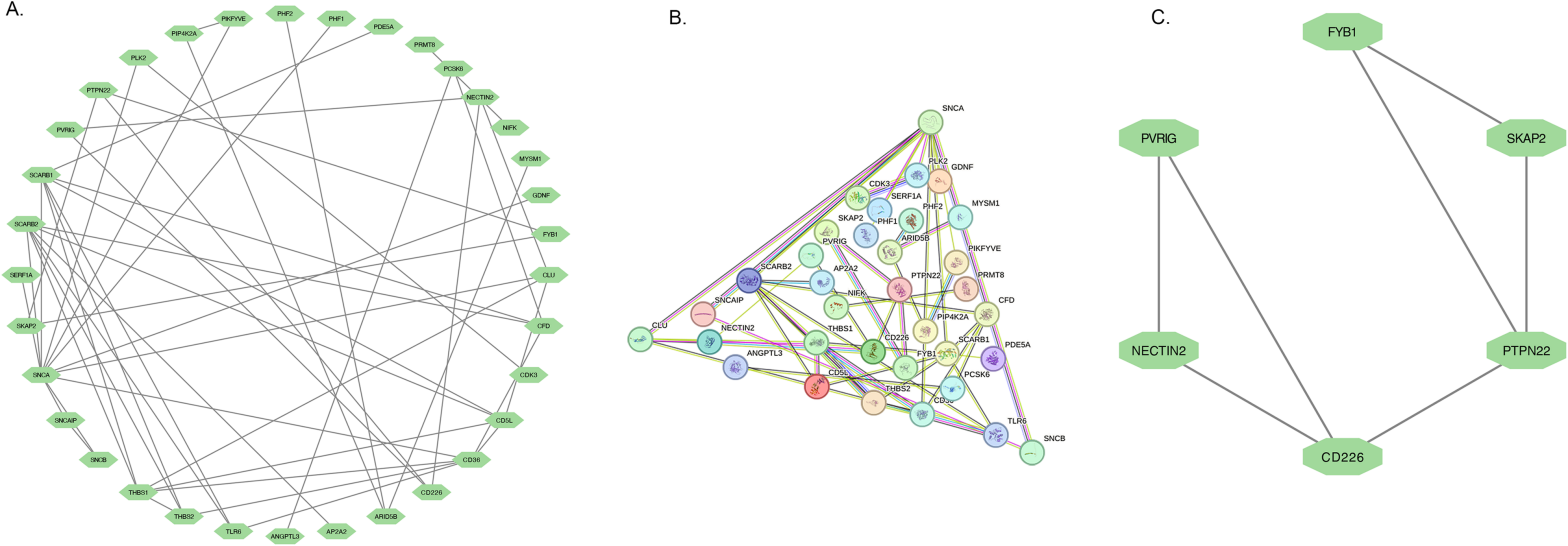
PPI network and the significant module of DEGs. **(A)** The significant module was obtained from PPI network constructed using Cytoscape with 34 nodes and 49 edges. **(B)** The PPI network of DEGs. **(C)** The 6 hub genes were analysed by Cytoscape MCODE.

A total of 25 DEGs were imported into the PPI network complex, comprising 34 nodes and 49 edges (**Figures 4A, B**). We then applied Cytotype MCODE for further analysis, and the results are shown in **Figure 4**. Six hub genes were identified in both diseases, and they were linked to cell adhesion molecules.

**Figure 5:**
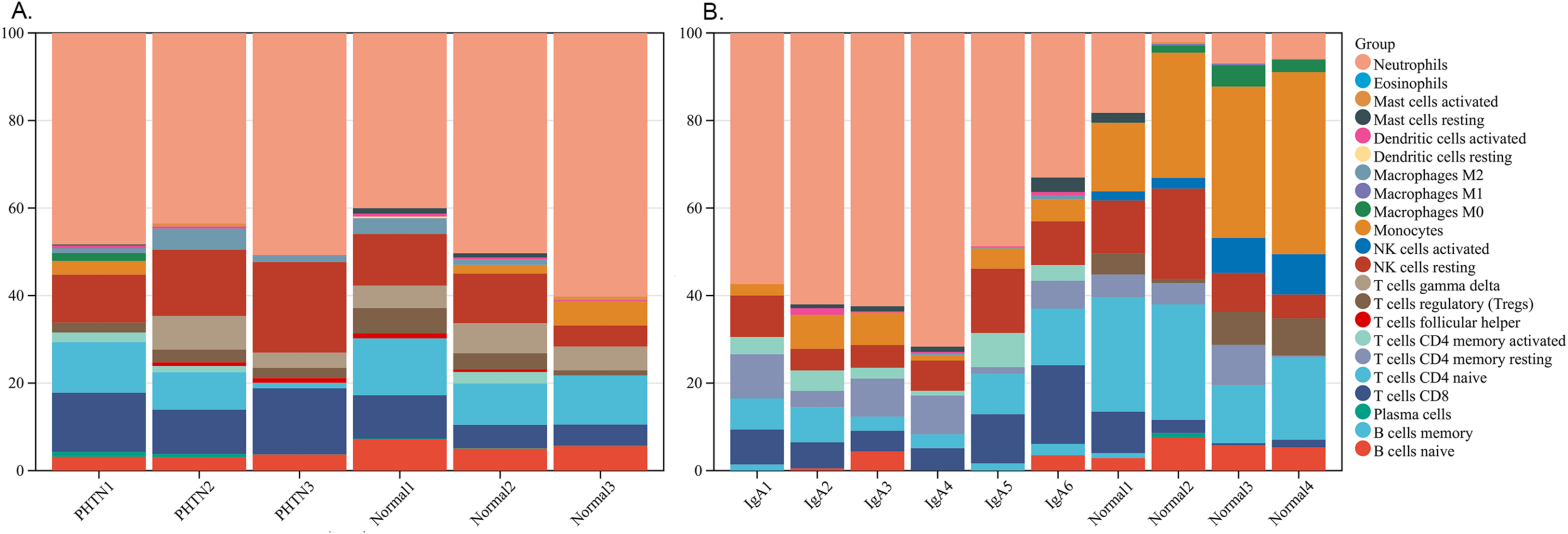
Stacked bar charts of 22 immune cell proportions. **(A)** PHTN, **(B)** IgA. Neutrophils accounted for the majority.

**Figure 6:**
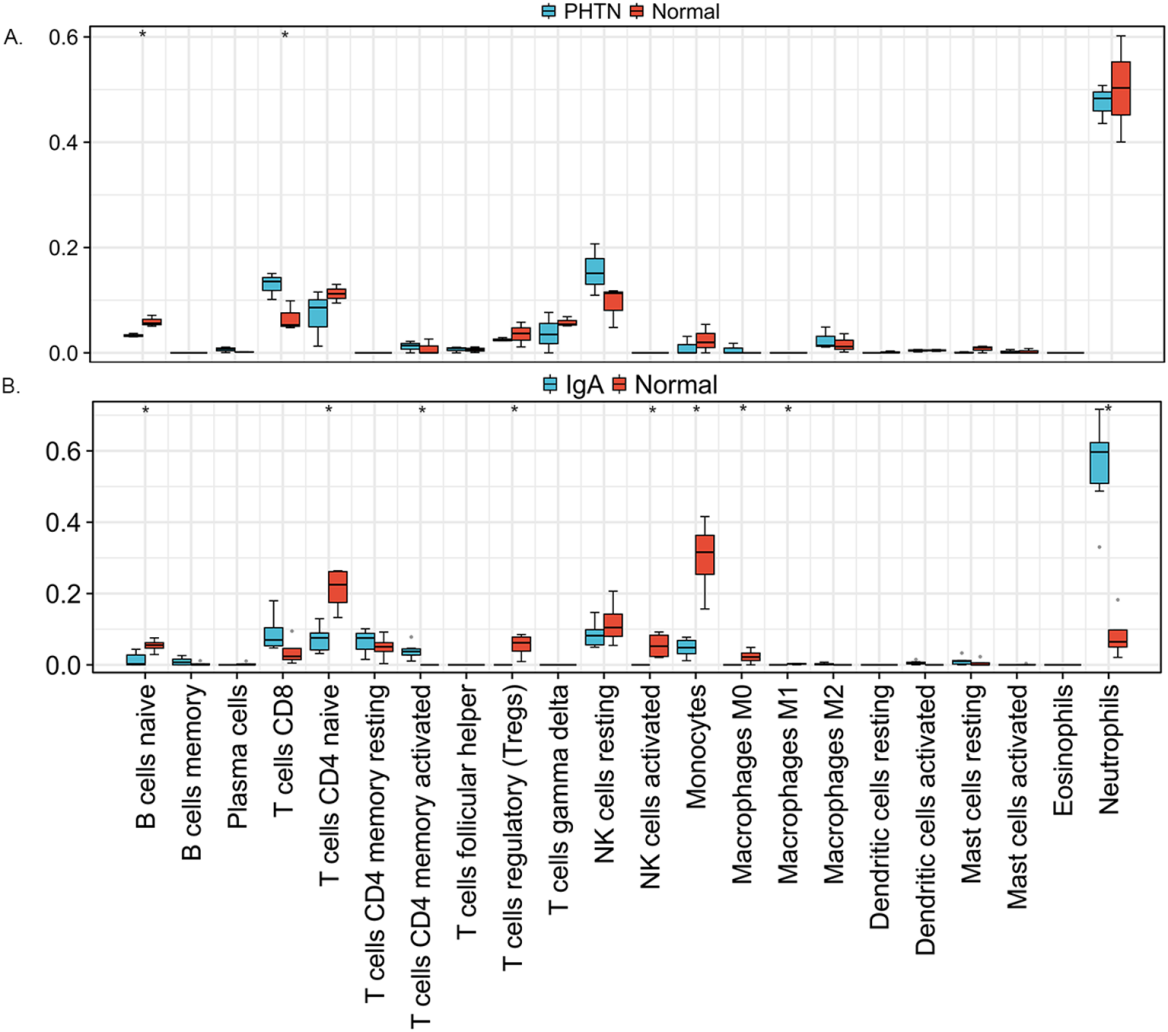
The differences of 22 immune cells. **(A)** PHTN, **(B)** IgA. *P < 0.05.

We used the online CIBERSORT algorithm to assess the expression changes in immune cells. We found that neutrophils accounted for the majority of all infiltrating cells (**Figure 5**). The differential expression proportion of immune-infiltrating cells in the PHTN and normal groups are B cell naïve (downregulated) and T cell CD4 (upregulated). We found that B cell naïve, T cell CD4 naïve, regulatory T cells (Tregs), activated NK cells, monocytes, macrophages M0 and M1 were downregulated. T cell CD4 memory activated and neutrophils were upregulated (**Figure 6**).

We have added this part to the manuscript and made corresponding revisions to the methods, results, and discussion sections.

### 3.6 Identification of bioactive compounds by CMap analysis

Given the role of 25 DEGs in PHTN and IgAV, we probed for potential therapeutic compounds that might best be suited to target these genes in order to achieve a beneficial therapeutic outcome. The top 10 compounds are shown in **Table 3**. These compounds sequentially ordered by score are: dicoumarol, tramiprosate, delivert, levetiracetam, troglitazone, salirasib, finasteride, febuxostat, metformin and dapagliflozin. Target genes corresponding to each compound were also listed. Together, these compounds and target genes provide a promising list for researchers or companies interested in conducting pre-clinical research into the mechanisms of and treatments for PHTN and IgAV.

**Table 3:** Top 10 compounds predicted to have activity against PHTN and IgAV as predicted via connectivity map.

ID	Cmap name	Dose	Cell	Score	Description	Target
BRD-A90547603	Eptifibatide	2.22 uM	JURKAT	-0.58	Platelet aggregation inhibitor	ITGA2B|ITGB3
BRD-K82236179	Dicoumarol	0.25 uM	JURKAT	-0.59	NADPH inhibitor	VKORC1|NQO1|CRYZ
BRD-K82234479	Tramiprosate	10 uM	JURKAT	-0.6	Beta amyloid inhibitor	APP
BRD-K49404994	Levetiracetam	0.74 uM	JURKAT	-0.61	Calcium channel blocker	SV2A|CACNA1B|SCN1A
BRD-A13084692	Troglitazone	10 uM	JURKAT	-0.62	PPAR receptor agonist|Insulin sensitizer	PPARG|AKR1B1|CCL2|CYP3A4|INS|ACSL4|ESRRA|ESRRG|SERPINE1|SLC29A1|TRPM3
BRD-K98453471	Salirasib	0.74 uM	JURKAT	-0.63	MTOR inhibitor	TRPA1|MTOR
BRD-A83081521	Finasteride	0.08 uM	JURKAT	-0.64	5-alpha reductase inhibitor	SRD5A2|SRD5A1|AR|AKR1D1
BRD-K48367671	Febuxostat	1.11 uM	JURKAT	-0.65	Xanthine oxidase inhibitor	XDH
BRD-K79602928	Metformin	0.25 uM	JURKAT	-0.69	Insulin sensitizer	INS|ACACB|PRKAB1
BRD-K46604138	Dapagliflozin	0.08 uM	JURKAT	-0.71	Sodium/glucose cotransporter inhibitor	SLC5A2|SLC5A1

### 3.7 Expression of fibronectin in kidney tissue

**Figure 7:**
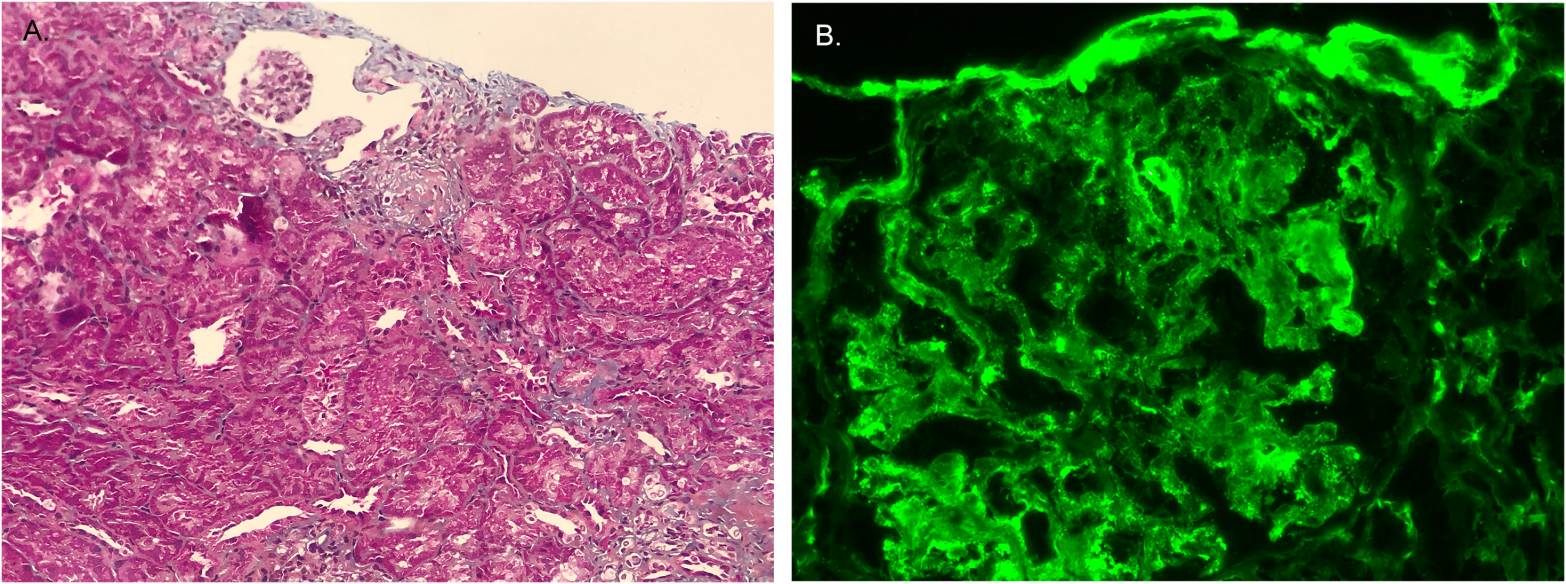
The expression of Fibronectin. **(A)** Fibronectin exhibits blue staining in Masson’s trichrome-stained renal tissue. **(B)** Immunofluorescence staining showed Fibronectin was positive.

Using immunofluorescence microscopy, we found that Fibronectin expression was markedly enhanced (**Figure 7**). Consistent with bioinformatics analysis results, Fibronectin expression was markedly elevated in hypertensive nephritis and IgA nephropathy.

## 4 Discussion

PHTN has become a major public health challenge, with prevalence rates rising to 3.5–5% in recent decades (16). Untreated PHTN predisposes children to long-term cardiovascular and renal complications, notably hypertensive nephropathy—a leading cause of progressive renal dysfunction and ESRD (17–19). IgAV/Henoch-Schönlein purpura, the most prevalent childhood small-vessel vasculitis (20), classically presents with palpable purpura, abdominal pain, arthralgia/arthritis, and renal involvement (IgAV nephritis, IgAVN) (21). Renal manifestations affect 20–54% of pediatric IgAV cases (22), ranging from self-limited hematuria to severe nephritis requiring intervention (23).

While PHTN and IgAV represent distinct entities, our case implies potential mechanistic overlaps. PHTN pathogenesis involves multifactorial interactions—genetic susceptibility, endothelial dysfunction (reduced nitric oxide bioavailability, elevated endothelin-1) (24), RAAS activation (25), sympathetic overactivity (26), sodium retention (27, 28), and chronic inflammation-driven vascular remodeling (29). In contrast, IgAV arises from IgA-dominant immune complex deposition triggered by infections or immune dysregulation (6, 30, 31), involving aberrant T-cell responses and elevated circulating IgA levels (32). These complexes deposit in small vessels, inciting inflammation across target organs (skin, gut, joints, kidneys) (33).

These 25 DEGs are PIP4K2A, MIER3, MYSM1, PDE5A, LMTK3, ZNF652, ERAP1, CFD, SESN3, AP2A2, CD36, ATF1, PCSK6, CHURC1, ZDHHC14, GRAPL, HSPC102, SNCA, PVRIG, NIFK, TBC1D10A, MBNL3, VWA5A, PHF2 and SKAP2. Through systematic literature review, we identified that among the 25 DEGs analyzed, CFD was the only gene with documented research relevance in IgAV. Current evidence highlights CFD as a critical mediator of alternative complement pathway activation in IgAVN, with study reporting significantly elevated urinary CFD levels in pediatric IgAVN patients compared to non-nephritis cases and healthy controls (34). This aligns with its proposed role in amplifying glomerular inflammation and proteinuria. In contrast, the remaining 24 DEGs lack direct experimental or clinical validation in IgAV and PHTN, underscoring a significant knowledge gap in their mechanistic or diagnostic relevance to these diseases. Further investigation is warranted to explore potential roles of these uncharacterized DEGs in IgAV and PHTN pathogenesis or progression.

Currently, there is a lack of research linking ECM receptor interactions to PHTN and IgAV. The ECM is a dynamic, three-dimensional network of macromolecules that provides structural and biochemical support to surrounding cells. Among its key components, fibronectin play a pivotal role in mediating interactions between cells and the ECM. Immunofluorescence staining revealed a significant increase in fibronectin, suggesting that ECM receptor interactions play an important role.

Immune infiltration analysis revealed that neutrophils exhibited the highest proportion in both PHTN and IgAV, while naive B cells were consistently downregulated. IgAV is characterized by IgA immune complex deposition and vascular inflammation, where neutrophil infiltration may serve as a hallmark of its acute phase. Hypertension-induced oxidative stress may activate intrarenal neutrophils, and intraglomerular hypertension directly damages endothelial cells, triggering neutrophil chemotaxis. In IgAV, antigens likely drive the differentiation of naive B cells into plasma cells, leading to a reduced proportion of naive B cells in peripheral blood. In PHTN, chronic inflammation may promote B cell differentiation, thereby depleting the naive B cell population. However, no direct supporting literature has been reported for these mechanisms, and further validation is required.

Effective PHTN management is critical to halting hypertensive nephropathy progression. Aggressive blood pressure control preserves renal function and mitigates cardiovascular risks in chronic kidney disease (CKD) (35). Untreated hypertensive nephropathy progresses to end-stage renal disease (ESRD); notably, proteinuria resolution in our case post-antihypertensive therapy highlights the urgency of early intervention to reduce glomerular hypertension and nephron loss (36). We identified compounds with potential dual efficacy against PHTN and IgAV: Eptifibatide: GPIIb/IIIa inhibitor (antiplatelet) (37). Unclear role in PHTN/IgAV. Dicoumarol: Anticoagulant with anti-inflammatory/renal perfusion benefits (38). Tramiprosate: Anti-inflammatory agent for IgAV and hypertension (39). Levetiracetam: Anticonvulsant with anti-inflammatory properties (40). Troglitazone: PPARγ agonist improving insulin sensitivity and renal protection (41). Salirasib: Ras inhibitor targeting inflammation/fibrosis pathways (42). Finasteride: Reduces proteinuria and preserves renal function (43). Febuxostat: Urate-lowering agent with antioxidative/anti-inflammatory effects (44). Metformin: AMPK activator attenuating kidney injury via metabolic modulation (45). Dapagliflozin: SGLT2 inhibitor lowering intraglomerular pressure and offering renoprotection (46).

While this case report provides valuable insights into potential shared molecular mechanisms between PHTN and IgAV, the inherent limitations of single-case study must be acknowledged. The conclusions drawn from a single patient may lack generalizability due to the unique genetic, environmental, and clinical factors influencing disease progression in individuals. Nevertheless, we hope this case report will offer clinicians a valuable perspective when encountering similar clinical presentations in their practice.

## 5 Conclusions

After BP-lowering agent treatment, the BP was controlled within the normal range, and urine protein turned negative. Renal pathology also showed significant improvement. It reminds us that aggressive BP-lowering agent was needed in PHTN. We used bioinformatics to determine the common DEGs between PHTN and IgAV. Six hub genes implicated in both diseases were linked to cell adhesion molecules. Moving forward, these shared hub genes could serve as novel targets for more in-depth mechanistic investigations and the development of fresh therapeutic interventions for individuals affected by PHTN and IgAV. Further research into these agents’ mechanisms of action will be crucial for optimizing treatment regimens and improving patient outcomes across these interconnected diseases.

## Data Availability

The datasets presented in this study can be found in online repositories. The names of the repository/repositories and accession number(s) can be found below: https://www.ncbi.nlm.nih.gov/, http://www.ncbi.nlm.nih.gov/geo.
